# A protocol of systematic review and meta-analysis of neuromuscular electrical stimulation for interstitial cystitis

**DOI:** 10.1097/MD.0000000000021088

**Published:** 2020-07-10

**Authors:** Da-yin Chen, Ying-xue Guo, Long-xin Dong, Wen-jie He, Hui-feng Cao, Ping Wang, Cai-fang Yue

**Affiliations:** a Department of Urology, The First Affiliated Hospital of Jiamusi University; b Department of Pharmacy, Jiamusi University; c Department of Outpatient, The First Affiliated Hospital of Jiamusi University; d Forensic Identification Center, Criminal Technology Division of Jiamusi Public Security Bureau; e Department of Critical Care Medicine, The First Affiliated Hospital of Jiamusi University, Jiamusi, China.

**Keywords:** effectiveness, interstitial cystitis, neuromuscular electrical stimulation, safety

## Abstract

**Background:**

This study will examine the effectiveness and safety of neuromuscular electrical stimulation (NMES) for the treatment of patients with interstitial cystitis (IC).

**Methods:**

We will retrieve the following electronic databases from their commencements to the March 1, 2020 to discover all related potential studies: MEDLINE, EMBASE, Cochrane Library, Web of Science, Cumulative Index to Nursing and Allied Health Literature (CINAHL), China National Knowledge Infrastructure, Chinese Biomedical Literature Database, Chinese Scientific Journal Database, and WANFANG Database. Randomized controlled trials related to the NMES for the treatment of patients with IC will be included, regardless publication status and language. Literature selection, data collection, and study quality assessment will be independently performed by 2 authors. The extracted data will be expressed as risk ratio and 95% confidence intervals for dichotomous data, and mean difference or standard mean difference and 95% confidence intervals for continuous data. RevMan V.5.3 software will be employed for statistical analysis.

**Results:**

This study will summarize current high quality randomized controlled trials to appraise the effectiveness and safety of NMES for the treatment of patients with IC.

**Conclusion:**

The findings of this study will provide helpful evidence to determine whether NMES is an effective treatment for patients with IC or not.

**Systematic review registration:**

PROSPERO CRD42020170495.

## 1 Introduction

Interstitial cystitis (IC) is a chronic, progressive debilitating bladder disorder,^[1–3]^ which is characterized by intermittent flares of frequency, urgent voiding, and pelvic pain.^[4–6]^ It is estimated that the prevalence rate of IC ranges from 0.3% to 2% according to clinical diagnosis around the world.^[7,8]^ The etiology of IC remains poorly understood.^[9]^ Despite several therapies are reported to manage IC, none of them can effectively treat this condition.^[10,11]^ Thus, it is very urgent to find potential candidates.

Fortunately, neuromuscular electrical stimulation (NMES) is recommended as a potential candidate and a variety of studies reported that NMES can be used to treat IC,^[12–26]^ which offers us new data for conducting this systematic review. Accordingly, this present study is designed to critically synthesize the most recent published data to appraise the effectiveness and safety of NMES for the treatment of patients with IC.

## 2 Methods

### 2.1 Study registration

This protocol has been registered on PROSPERO (CRD42020170495), and it is reported in accordance with the Preferred Reporting Items for Systematic Reviews and Meta-Analysis (PRISRMA) Protocol statement guidelines.^[27,28]^

### 2.2 Ethics and dissemination

No individual data will be extracted, thus no ethical approval is required. This study will be disseminated through a peer-reviewed journal or a conference meeting.

### 2.3 Study eligibility criteria

#### 2.3.1 Types of studies

Without limitations on language and publication status, this study will include randomized controlled trials that explore the effectiveness and safety of NMES for the treatment of patients with IC.

#### 2.3.2 Types of participants

All patients who were diagnosed as IC will be included, regardless gender, race, age, economic status, duration and severity of IC.

#### 2.3.3 Types of interventions

##### 2.3.3.1 Experimental interventions

All patients in the experimental group were treated with NMES only. Any treatments combined with NMES will be excluded.

##### 2.3.3.2 Control interventions

All patients in the control group were treated with any interventions, such as oral medication, moxibustion, and physical therapy. However, any management combined with NMES will be excluded.

##### 2.3.3.3 Type of outcome measurements

Primary outcomes are pain intensity (as measured by Visual Analog Scale or other pain scales), and improvement of overall symptoms (as assessed by patient-reported global response assessment or other tools).

Secondary outcomes are urinary frequency episodes, quality of life (as checked by 36-Item Short Form Survey or other questionnaires), and adverse events.

### 2.4 Search strategy and data management

#### 2.4.1 Search strategy

The electronic databases will be comprehensively retrieved from their commencements to the March 1, 2020 to identify all related potential studies: MEDLINE, EMBASE, Cochrane Library, Web of Science, Cumulative Index to Nursing and Allied Health Literature (CINAHL), China National Knowledge Infrastructure, Chinese Biomedical Literature Database, Chinese Scientific Journal Database, and WANFANG Database. There are no limitations to publication language and status. A search strategy for MEDLINE has been established (Table 1). Identical search strategies will be applied to all other electronic databases.

**Table 1:**
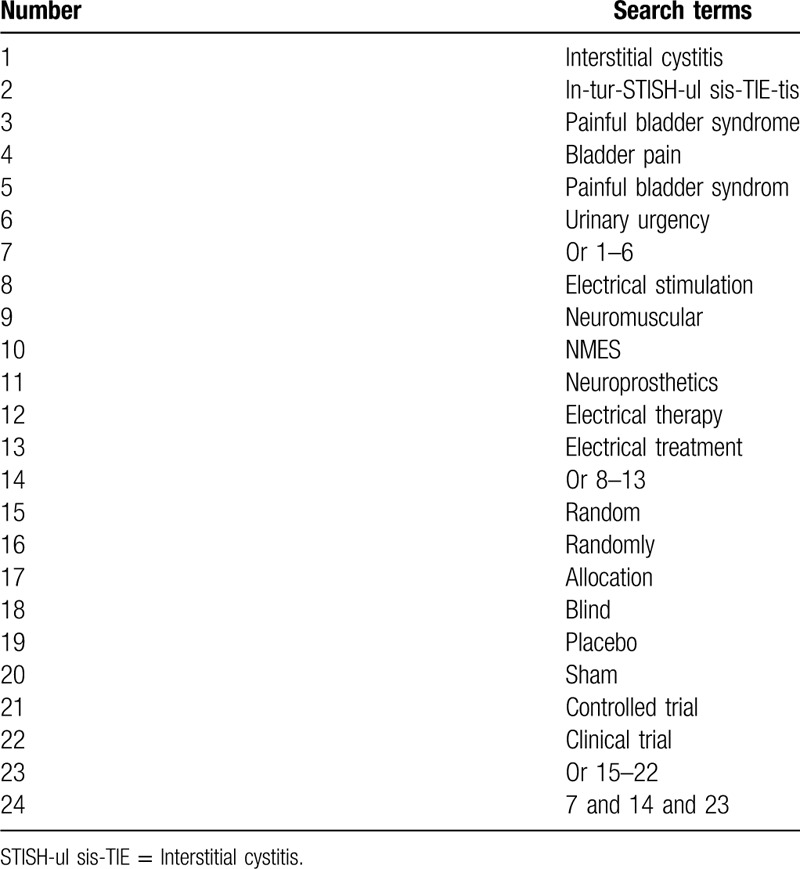
Search strategy used in MEDLINE database.

We will also search relevant conference abstracts, clinical trial registries for ongoing trials, and reference lists of all related reviews.

#### 2.4.2 Study selection

Two authors will independently import all citations into EndNote X9 to eliminate duplicated ones. Titles/abstracts of all potential records will be screened to remove any irrelevant studies. If necessary, we will obtain and read full-text of remaining literatures according to the eligibility criteria. All excluded studies will be noted and summarized with reasons. Any differences will be worked out with the help of another author and a consensus will be made. The results of study selection will be summarized in a PRISRMA flow diagram.

#### 2.4.3 Data extraction and management

Two authors will independently extract data by a standardized data form developed specifically for this study. Any confusion will be cleared up with the help of another author and a final conclusion will be made. The information includes study general information (eg, title, first author, year of publication), participant characteristics (age, gender), diagnostic criteria, inclusion and exclusion criteria, study design, sample size, interventions, outcomes, results, findings, adverse events, and funding information.

#### 2.4.4 Dealing with missing data

Any insufficient or missing data will be requested from primary authors. If they can not provide those data, we will perform data analysis based on the available data, and we will discuss its affects on the study findings.

### 2.5 Study quality assessment

The Cochrane Collaboration's tool will be utilized to assess study quality of included trials by 2 independent authors. Each study will be identified at 7 aspects, and each 1 is graded as 3 levels: low, unclear, and high risk of bias. Divergences will be arbitrated with the help of another author.

### 2.6 Statistical analysis

#### 2.6.1 Data synthesis

RevMan 5.3 software will be utilized for statistical analysis. Risk ratio with 95% confidence intervals will be used to measure the treatment effect for dichotomous outcome data. Mean difference or standardized mean difference and 95% confidence intervals will be suggested to measure the treatment effect for continuous outcome data. Statistical heterogeneity will be examined by *I*^*2*^ test. *I*^*2*^ ≤ 50% suggests homogeneity, and we will use a fixed-effect model. If possible, we will conduct a meta-analysis when sufficient trials are included. *I*^*2*^ >50% indicates considerable heterogeneity, and we will place a random-effect model. In addition, we will perform subgroup analysis to check sources of obvious heterogeneity. If there is still remarkable heterogeneity after subgroup analysis, a meta-analysis will not be carried out. However, we will report outcome results using a narrative synthesis.

#### 2.6.2 Subgroup analysis

If studies are adequate, we will perform a subgroup analysis based on the different types of interventions, controls, and outcome measurements.

#### 2.6.3 Sensitivity analysis

Whenever it is appropriate, we will undertake a sensitivity analysis to test stability of study findings by removing low quality studies, or small sample studies.

#### 2.6.4 Reporting bias

We will adopt a funnel plot and Egger's regression test to investigate reporting bias if 10 or more trials are included.^[29,30]^

#### 2.6.5 Grading the quality of evidence

Two authors will independently appraise quality of evidence for each outcome by Grading of Recommendations Assessment, Development and Evaluation, which grades quality of evidence as 4 levels: high, moderate, low, and very low.^[31]^ Any uncertainty will be solved with the help of an independent arbitrator.

## 3 Discussion

Some studies have shown that NMES may benefit patients with IC.^[12–26]^ However, the results are still not consistent, and so far no systematic review of NMES in treating IC has been identified. Thus, this study intends to present the merged data and to carry out a systematic review of NMES for IC in order to supply high-quality evidence. Its results may provide reference and recommendation for clinician and scientific searchers.

## Author contributions

**Conceptualization:** Da-yin Chen, Ying-xue Guo, Long-xin Dong, Wen-jie He, Hui-feng Cao, Ping Wang, Cai-fang Yue.

**Data curation:** Da-yin Chen, Long-xin Dong, Wen-jie He, Hui-feng Cao, Ping Wang, Cai-fang Yue.

**Formal analysis:** Da-yin Chen, Ping Wang.

**Funding acquisition:** Cai-fang Yue.

**Investigation:** Cai-fang Yue.

**Methodology:** Da-yin Chen, Ying-xue Guo, Wen-jie He, Hui-feng Cao, Ping Wang.

**Project administration:** Cai-fang Yue.

**Resources:** Da-yin Chen, Ying-xue Guo, Long-xin Dong, Wen-jie He, Hui-feng Cao, Ping Wang.

**Software:** Da-yin Chen, Ying-xue Guo, Long-xin Dong, Wen-jie He, Hui-feng Cao, Ping Wang.

**Supervision:** Cai-fang Yue.

**Validation:** Da-yin Chen, Ying-xue Guo, Long-xin Dong, Ping Wang, Cai-fang Yue.

**Visualization:** Da-yin Chen, Long-xin Dong, Hui-feng Cao, Ping Wang, Cai-fang Yue.

**Writing – original draft:** Da-yin Chen, Ying-xue Guo, Long-xin Dong, Wen-jie He, Hui-feng Cao, Ping Wang, Cai-fang Yue.

**Writing – review & editing:** Da-yin Chen, Ying-xue Guo, Wen-jie He, Ping Wang, Cai-fang Yue.
